# Optimal Agents for Visualizing Collagen Tissue Microarchitecture Using Contrast-Enhanced MicroCT

**DOI:** 10.3390/ph16121719

**Published:** 2023-12-12

**Authors:** Spencer B. Glancy, Herman Douglas Morris, Vincent B. Ho, George J. Klarmann

**Affiliations:** 1 San Antonio Uniformed Services Health Education Consortium, San Antonio, TX 78234, USA; spencer.b.glancy.mil@health.mil; 2 School of Medicine, Uniformed Services University, Bethesda, MD 20814, USA; herman.morris@usuhs.edu (H.D.M.); vincent.ho@usuhs.edu (V.B.H.); 3 4D Bio3 Center for Biotechnology, Uniformed Services University, Bethesda, MD 20814, USA; 4 The Geneva Foundation, Tacoma, WA 98402, USA

**Keywords:** MicroCT, contrast agent, collagen, Lugol’s, phosphotungstic acid

## Abstract

Micro-computed tomography (microCT) is a common tool for the visualization of the internal composition of organic tissues. Collagen comprises approximately 25–35% of the whole-body protein content in mammals, and the structure and arrangement of collagen fibers contribute significantly to the integrity of tissues. Collagen type I is also frequently used as a key structural component in tissue-engineered and bioprinted tissues. However, the imaging of collagenous tissues is limited by their inherently low X-ray attenuation, which makes them indistinguishable from most other soft tissues. An imaging contrast agent that selectively alters X-ray attenuation is thus essential to properly visualize collagenous tissue using a standard X-ray tube microCT scanner. This review compares various contrast-enhanced techniques reported in the literature for MicroCT visualization of collagen-based tissues. An ideal microCT contrast agent would meet the following criteria: (1) it diffuses through the tissue quickly; (2) it does not deform or impair the object being imaged; and (3) it provides sufficient image contrast for reliable visualization of the orientation of individual fibers within the collagen network. The relative benefits and disadvantages of each method are discussed. Lugol’s solution (I_3_K), phosphotungstic acid (H_3_PW_12_O_40_), mercury(II) chloride (HgCl_2_), and Wells–Dawson polyoxometalates came closest to fitting the criteria. While none of the contrast agents discussed in the literature met all criteria, each one has advantages to consider in the context of specific lab capabilities and imaging priorities.

## 1. Introduction

**Figure 1:**
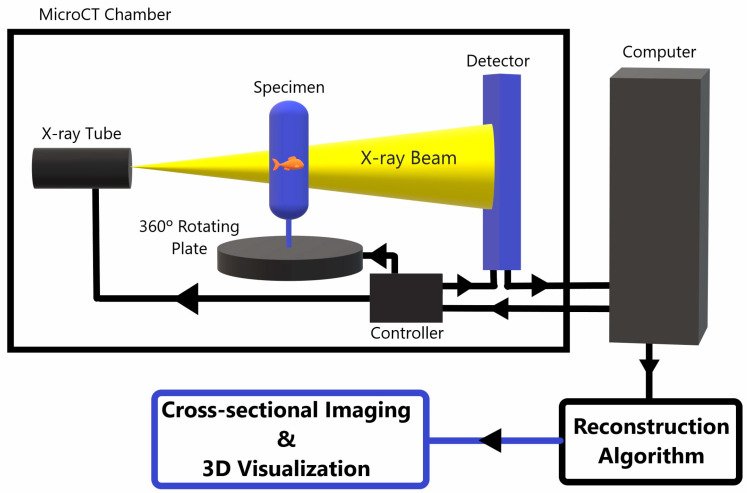
A schematic diagram of a standard X-ray tube MicroCT scanner. The specimen is placed between an X-ray source and a detector. The specimen rotates 360° while the X-ray tube and detector stay fixed within the scanner. The detector picks up the 2D X-ray penetration patters at each rotational position and sends the data to a computer, which processes the 2D data from each rotational view to tomographically reconstruct the 2D X-ray data into a 3D microCT image data set. This data can be post-processed and reformatted into various projections for improved visualization of the internal composition of the imaged object [1,2,3,4].

MicroCT can analyze biological tissues at high resolution on a microscopic level. It relies on the use of X-rays for three-dimensional (3D) imaging, and serves as an important tool for analyzing tissue specimens or bioengineered tissue, as it allows for volumetric visualization and provides a key supplement to microscopic optical imaging. Figure 1 shows a basic schematic of a standard table X-ray tube microCT scanner.

Like microscopic visualization of histochemical staining, microCT has the potential to analyze biological tissues at high resolution on a microscopic level, with the advantages of three-dimensional tissue imaging, and without the need to physically cut up the specimen. However, there are relatively limited data available regarding the effectiveness of contrast-enhancing agents in microCT [5]. Most of the contrast agents that have been tested are the same or are similar to agents already used in histological staining or macroscopic contrast-enhanced CT (CE-CT) given their wide commercial availability, relatively low cost, familiarity among researchers and clinicians, and known binding properties. Some have been shown to be quite effective with CE-microCT as well. There are also new contrast agents being developed for microCT based on the combined knowledge of X-ray attenuation, biophysics, and molecular binding properties. All of these will be discussed later in this review. 

Variations in how X-rays interact with different constituents of an object, called attenuation, produce differences in image contrast that enable visualization of more complex structural densities [6,7]. High-density materials composed of high atomic number elements will attenuate more X-rays. The normal X-ray spectrum used for imaging generally consists of a range of photon energies. Constituents of the object with different densities and atomic numbers will attenuate the X-ray beam differently, yielding a depiction of their interface in the resultant image [6,7]. Thus, for example, bone (high density) shows up more clearly on an X-ray film compared to soft tissues (low density) because while a high energy X-ray can pass straight through bone, a lower energy X-ray is significantly absorbed or attenuated. For the same reasons, however, homogenous materials of similar densities and with similar linear attenuation coefficients can be difficult to resolve with CT, especially on a microscopic scale. Various methods—including the use of contrast enhancing agents, phase-contrast imaging, and high-coherence synchrotron-generated X-rays—have been used to improve contrast resolution in homogenous materials such as soft tissues. 

In addition to tube-generated X-rays used in scanners such as the one modeled in Figure 1, X-rays for CT imaging can also be generated using synchrotron radiation. Synchrotron-generated X-rays have a much higher intensity and flux compared to standard tube-generated X-rays. This creates a more brilliant image and allows for greater tunability of the photon energy to the optimum value for the investigated sample. Synchrotron-generated X-rays are also highly collimated, almost-parallel beams which causes reduced scatter, increased signal-to-noise ratio, and therefore also has improved contrast resolution [8]. Unfortunately, synchrotrons are limited by their need for a very large footprint, making them unconventional for general laboratory-based research at the time of writing this article. Synchrotron radiation CT has been proven to be a very effective way to increase contrast resolution within soft tissues [9]. However, this review focuses more specifically on how contrast can be enhanced with more widely available microCT systems, which utilize tube-generated X-rays. 

One of the most common methods to improve X-ray contrast resolution is to add a contrast agent. A contrast agent improves resolution by filling in or diffusing through an area of interest, altering its ability to attenuate X-rays, and highlighting it in relation to surrounding structures [6,10]. It can be either positively attenuating (e.g., iodine or barium which have a higher attenuation relative to soft tissue) or negatively attenuating (e.g., low atomic number compounds such as gases or lipids). Many different staining techniques have been developed, each designed to produce maximum contrast of the target of choice within a wide array of tissue types. In clinical medicine, contrast agents are used to visualize vascular anatomy, organ morphology, or gastrointestinal luminal details [10]. Contrast-enhancing staining agents (CESAs) are the predominant agents used for microCT, where they are chosen for their ability to bind to a specific target, causing it to stand out in relation to the surrounding structure or tissues so that the whole can be visualized in greater detail [2].

Soft tissues are among the most difficult to visualize using X-ray tomography, as many components of soft tissues other than fat—such as muscle, ligaments, fascia, and blood vessels—have similar X-ray attenuation properties [2]. For example, a good CESA can be one that is able to exclusively bind to the soft tissue components of interest and alter their X-ray attenuation in a way that provides greater definition and resolution with microCT. The primary difficulty is developing or finding a contrast agent specific enough to target and highlight, and thus visualizing the microstructure of a targeted soft tissue component. To do so, the contrast agent must exclusively bind to or outline the molecules that make up that microstructure. Within the soft tissues of the body, one of the most important and prevalent of these molecules may be collagen. 

Collagen Type I is an important structural protein, making up 25–35% of the whole-body protein content in mammals [11]. The arrangement of collagen fibers contributes significantly to the structural integrity of various organs found throughout the body due to their impressive tensile strength [11]. Collagen is also useful as a marker for tissue organization. Not surprisingly, it is an important component of connective tissue. Being able to understand how a collagen fiber arrangement correlates with its function and integrity within the connective tissue would help with understanding the pattern of collagen breakdown, both spontaneously and as a reaction to various forms of mechanical stress. Developing this understanding could then open the way for innovative ways to characterize soft tissue injuries based on microscopic findings. A more detailed understanding of collagen microarchitecture could also be used to help with the creation of collagen-based bio-prostheses that emulate human physiology and anatomy not only on a functional macroscopic level but a microscopic level as well. Furthermore, microCT imaging following the fabrication of tissues 3D printed with collagen-rich bioink may help inform us on how the microstructure compares to actual tissue architecture. MicroCT examination of the 3D-printed tissues post-implantation in animals would provide information regarding tissue integration and durability, and help guide redesigns of the tissue-engineered constructs if necessary. 

The ability to visualize collagen microarchitecture requires not only that the collagen fibers themselves be differentiated from surrounding structures (i.e., contrast resolution, as described above), but also be a small enough pixel size (i.e., spatial resolution) to differentiate between two adjacent fibers. Depending on the type and density of tissue being studied, the resolution of collagen fibers requires a voxel size on the scale of 0.5 to 25 μm. Several microCT (and nanoCT) scanners are commercially available which have the means of achieving a sufficiently small voxel size for collagen microarchitecture. Unfortunately, despite the ability of these scanners to achieve a high spatial resolution, without a way to improve contrast resolution or the signal-to-noise ratio, the precise three-dimensional arrangement and orientation of these fibers is difficult to visualize using a standard microCT scanner. However, with the right contrast agent—as combined with sound tissue preparation and scanning techniques—sufficient resolution of individual collagen fibers can be achieved.

This review compares various techniques used and reported in the literature for visualizing the microarchitecture of collagen-based tissues using microCT, with the goal of identifying the optimal CESA for visualizing the 3D arrangement of collagen fibers within soft tissues using contrast-enhanced microCT in an ex vivo setting using an X-ray tube microCT scanner. An ideal CESA would meet the following criteria: 1. It diffuses through tissue quickly; 2. It does not deform the subject being imaged, and 3. It provides sufficient contrast resolution to visualize the orientation of individual fibers within the collagen network.

This review builds on prior work comparing various CESAs used in microCT. Pauwels et al. compared the effectiveness of several different contrast agents by staining bacon (adipose and muscle) and the hind legs of mice (muscle, fat, bone, cartilage, and tendon) [12]. De Bournonville et al. also conducted a literature review comparing contrast agents used for various musculoskeletal tissues (cartilage, bone marrow, muscle, tendons, and ligaments) [13]. These reviews provide an excellent comparison of many of the common contrast agents used today. This review seeks to add a more detailed focus on collagen specifically, extracting information from the available literature to learn how to best visualize collagen’s fine microarchitecture with CE-microCT. 

## 2. Results 

The 48 contrast agents can be categorized based on their ionization and molecular structure (Table 1). The categories include ionic iodinated, nonionic iodinated, gadolinium-based, polyoxometalates (POMs), or other metallic compounds [13]. These characteristics are the most relevant in terms of what gives the contrast agent its ability to bind target molecules and attenuate X-rays. 

Ionic iodinated compounds have been seen to work well as contrast agents for some collagen-containing tissues such as cartilage. However, they provide contrast due to their interaction with negatively charged glycosaminoglycans (GAGs) within the tissues rather than inert, water-insoluble collagen fibers. Most of the articles detailing the use of these compounds commented on the tissue’s GAG content, but had little, if anything, to conclude about collagen [13,14,15]. The most used of these compounds in the reviewed literature is Lugol’s solution (I_3_K). Lugol’s primary advantage over other CESAs is its relatively quick diffusion time due to its small size and water solubility [14]. Balint et al. stained porcine tendons and ligaments with Lugol’s, phosphotungstic acid (PTA, H_3_PW_12_O_40_), and phosphomolybdic acid (PMA, H_3_PMo_12_O_40_). MicroCT showed that Lugol’s solution took <1 day to diffuse to the center of a porcine anterior cruciate ligament, whereas PTA and PMA took approximately 5 days (~1–2 mm/day) [16]. However, Lugol’s solution was not shown to provide sufficient contrast enhancement to see collagen fibers at a voxel size of 7.5 µm. Although it did diffuse quickly through the collagenous tissue, it did not selectively bind to it, since it stained other constituents of the tissue as well. Therefore, the contrast was only mildly improved even when scanned with a voxel size of 1 µm [13,17,18]. Based on the reviewed articles, Lugol’s solution and other ionic iodinated compounds would not be considered optimal for achieving the micrometer-scale contrast resolution required to study collagen microarchitecture, although it is useful for allowing visualization of tissue organization on a larger scale. 

Gadolinium-based agents have become the standard CESA for use in magnetic resonance imaging (MRI), as these agents are paramagnetic and can be detected on MRI. However, the density of Gadolinium-based agents also attenuates X-rays and can serve as a CESA for microCT as well. As with ionic iodinated compounds, gadolinium-based CESAs have been shown to increase contrast resolution with the microCT of proteoglycan-rich tissues, but little to nothing is written in the reviewed literature about the visualization of collagen networks specifically [13,19]. Gadolinium-based CESAs were able to diffuse through bovine nasal cartilage disks of 6 mm diameter and 1 mm thick after being soaked for 24 h [19]. However, the scans were performed at a voxel size of 25 µm, the upper limit of voxel size for resolving collagen fibers. Although no singular studies were found directly comparing PTA and gadolinium-based CESAs, the diffusion rate of gadolinium-based compounds appears to be faster than that of PTA based on a comparison of multiple studies. 

**Figure 2:**
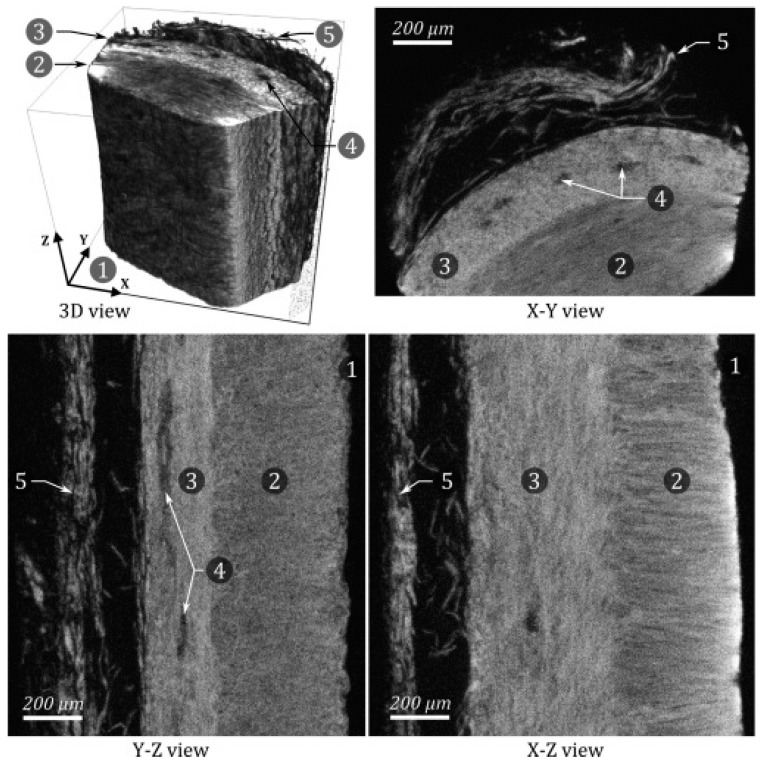
Different MicroCT views of a small sample of PTA-stained porcine vein wall. 1. Lumen of the vein. 2. Media. 3. Adventitia. 4. Vasa vasorum. 5. Surrounding conjunctive tissue. Note how sufficient resolution is achieved to visualize that the collagen fibers in the media lie perpendicular to the fibers that comprise the adventitia. It should be noted that while the direction of the collagen fibers can be inferred from the image, what the image actually shows is a moiré interference pattern caused by the overlapping and close proximity of the near parallel fibers, and not the fibers themselves. Reprinted/adapted with permission from Ref [17]. Copyright © 2023 Academié des sciences. Published by Elsevier Masson SAS.

POMs are polyatomic ions that typically contain three or more transition metals linked by oxygen atoms to form sophisticated three-dimensional networks. The high atomic number and electron density of these compounds gives them strong X-ray attenuation properties [20]. The most widely used POMs for biomedical purposes are (PTA) and (PMA). These compounds are used for histochemical staining processes due to their ability to selectively bind fibrin, collagen, and other connective tissue fibers. PTA and PMA have a relatively high negative charge density and a higher volume fraction of metal to molecular weight ratio compared to other CESAs. These properties give PTA and PMA a strong affinity for collagen, which has a positive net charge in low pH [21,22]. As discussed above, this combination of being able to selectively bind collagen and to allow increased X-ray attenuation once bound is the foundation of an effective CESA. PTA was the most used CESA for staining soft tissues in the reviewed literature due to its ability to significantly improve the image contrast resolution of collagen. Nierenberger et al. reported that PTA allowed enough image contrast to see the three-dimensional arrangement of collagen fibers in the walls of porcine veins, as demonstrated in Figure 2 (scanned at a voxel size of 1 µm) [17].

The significant downsides of PTA and PMA are that they are highly acidic, causing protein denaturation and tissue breakdown, and that they diffuse through tissues relatively slowly, as noted above, which makes them difficult to use in larger specimens. Multiple studies have shown that tissues stained with PTA and PMA deform and degrade significantly over the course of just several days, with as much as 20% inhomogeneous shrinkage [16]. However, a study by Missbach-Guentner et al. reported little to no tissue distortion by PTA in the staining of murine kidneys when using their sample preparation method [23]. This could be due to the differing effects of PTA on kidneys vs. the more fibrous tissues tested in other articles, or to Missbach-Guentner et al.’s more gradual, stepwise approach to dehydrating the tissue specimens before adding the CESA, followed by a gradual rehydration process before scanning [23,24]. More investigation is needed to understand these differences. Other methods for stabilizing tissue with hydrogel during preparation for staining solvent conditions have been discussed by Wong et. al. through using the low-molecular-weight Lugol’s (EtOH) stain which is equally applicable to POMs agents [25].

Other lesser-known POMs have also been tested with microCT. De Clercq et al., and Kerckhofs performed microCT studies using different isomers and metal substitutions of Wells–Dawson POMs (WD-POMs) at voxel sizes of 7 µm and 2 µm respectively [26,27,28]. Unlike the other CESAs discussed, these molecules are not commercially available, and must be synthesized before use [29,30]. The WD POMs can be dissolved in a phosphate buffer solution at or close to physiological pH, thus significantly reducing tissue destruction compared with PTA and PMA. Of the WD POMs tested, Monolacunary WD POM (Mono-WD POM), Parent-WD POM, and Hafnium-substituted WD POM (Hf-WD POM) offer about the same contrast resolution enhancement compared to PTA in the staining of kidney and long bone tissue [26].

Mono-WD POM and Hf-WD POM were shown to have the fastest diffusion rates compared to all other POMs. Parent WD-POM and PTA failed to diffuse all the way to the core of a murine kidney after 4 days, while the other POMs used in the study were able to do so [26]. Mono-WD POM is an intermediate in the synthesis of Hf-WD POM, so its synthesis is more cost-effective and easier. With this in mind, Mono-WD POM appears to be one of the most promising CESAs found in the literature for staining soft tissues for CE-MicroCT if it can be synthesized. Its contrast enhancement and diffusion rate were improved even further when LiCl was added to the staining solution [26]. Kerckhofs et al. confirmed Hf-POM and PTA have a strong binding affinity for collagen I, II, and fibrin using Raman spectroscopy, which showed an average drop of 28% in the peak intensity ratio between the POM peak and protein peak before and after rinsing of the tissue samples [28]. While Mono-WD POM was not tested for its binding affinity for collagen specifically, given its similar molecular structure and characteristics, it may have a similar affinity. Thus, further study is warranted.

Many other metallic compounds have also been tested as microCT CESAs for collagenous soft tissues [12,13,31]. Pawels et al. tested the binding affinity of various CESAs for connective tissue vs. adipose vs. muscle, as well as their diffusion rates and whether the CESAs would remain fixed in the tissue samples over time and after rinsing. Pauwels et al. performed their scans at voxel sizes ranging from 23 to 40 µm, in the upper range of voxel size needed to resolve collagen fibers. Therefore, more data using a smaller voxel size would be helpful to draw a more definitive conclusion on their ability to highlight collagen specifically, but they found binding properties which show promising results. Their comparison showed that iron(III) chloride and sodium tungstate stained connective tissue more than muscle. Ammonium molybdate, mercury(II) chloride, sodium tungstate, lead nitrate, barium nitrate, and barium chlorate provided the best visualization of tendons under microCT. Individual muscle fascicles were best visualized with PMA, PTA, and mercury(II) chloride. The only CESAs that remained fixed in the tissue samples after leaving the stained samples in water for 4 days were mercury(II) chloride, PTA, PMA, and ammonium orthomolybdate. Based on these conclusions, mercury(II) chloride should also be considered an option, as it is relatively inexpensive compared to the POMs and may provide sufficient resolution under the right preparation and scanning conditions. Unfortunately, like PTA and PMA, it diffuses relatively slowly through tissues, is highly toxic, and generates hazardous waste, which can be difficult and expensive to dispose. Sodium tungstate is another potential option, as tungsten has a high binding affinity for collagen and diffuses more easily through tissues than many of the other CESAs mentioned above.

**Table 1:** Comparison of contrast-enhancing staining agents used on collagen-containing soft tissues.

CESA	Proven to Show Collagen Fiber Arrangement?	Tissue Distortion	Diffusion Time	Comments
Anionic Iodinated				
Ioxaglate (C_24_H_2I_I_6_N_5_O_8_), Iothalamate (C_11_H_8_I_3_N_2_O_4_) [13,15]	No	No data	Slow	Studied using a voxel size of 12 µm. Large quantities required, improve contrast in GAG-rich tissues (cartilage).
Lugol’s (I_3_K) [1,12,13,16,17,32,33]	Limited	Yes	Fast	Studied using a voxel size of 1 to 7.5 µm. Increases attenuation of collagen. However, it also stains other constituents of tissue, only mildly increasing contrast resolution.
Cationic Iodinated				
CA^4+^, CA^1+^, CA^2+^ [13,14]	No	Requires further study	Fast through GAG-rich or anionic tissues	Studied using a voxel size of 30 µm. Higher positive charge correlates with a higher attenuation.
Nonionic Iodinated				
Itopride (C_18_H_24_I_3_N_3_O_8_), Iodixanol (C_35_H_44_I_6_N_6_O_15_), Iomeprol (C_17_H_22_I_3_N_3_O_8_) [13,14]	No	No data	Slow	Studied using a voxel size of 30 µm. Pharmaceutical radiocontrast that partitions due to MW or hydration.
Gadolinium				
Gadopentetate (dimeglumine, C_28_H_54_GdN_5_O_20_), Gadoteridol (C_17_H_29_GdN_4_O_7_), Gd^3+^ [13,19]	Requires further study	Requires further study	Faster than PTA	Studied using a voxel size of 25 µm. Effectiveness as a CESA directly correlates with proteoglycan content of tissue.
POMs				
PTA (H_3_PW_12_O_40_) [12,13,16,17,21,23,24,26,28,34,35,36,37,38,39] PMA (H_3_PMo_12_O_40_) [12,13,16,17]	Yes	Yes	Slow	Studied using voxel size of 1 to 40 µm. PTA is a known histochemical staining agent for binding collagen.
Zr-POM ((Et_2_NH_2_)_10_[Zr(Pw_11_O_39_)_2_]·7H_2_O) [27] 1:2 Hafnium (IV) substituted WD POM (K_16_[Hf(*a*_2_-P_2_W_17_O_61_)_2_]·19H_2_O) [26,28] Parent WD POM (*a-/b*-K_6_P_2_W_18_O_62_·14/19H_2_O) [26] Monolacunary WD POM (*a_2_*-K_10_P_2_W_17_O_61_·20H_2_O) [26] Trilacunary WD POM (Na_12_[*a*-P_2_W_15_O_56_]·24H_2_O) [26]	Yes	No	Slow to Fast	Studied using voxel size of 2 to 7 µm. High binding affinity for collagen. Hf-WD POM, parent WD POM, and Mono-WD POM shown to provide sufficient contrast for visualizing collagen fibers. Hf-WD POM and Mono-WD POM have faster diffusion rates than PTA. Not available commercially, must be synthesized in the lab. Of these options, Mono-WD POM is more cost-effective to synthesize
Other Metallic Compounds [12,13]	Comments			
FeCl_3_ (NH_4_)_2_MoO_4_ HgCl_2_ Na_2_WO_4_ Ba(ClO_3_)_2_ Ba (NO_3_)_2_ Pb (NO_3_)_2_	Studied using a voxel size ranging from 23 µm to 40 µm. (Iron (III) chloride and sodium tungstate stained connective tissue more than muscle. Ammonium molybdate, (mercury (II) chloride, sodium tungstate, lead nitrate, barium nitrate, and barium chlorate provided the best visualization of tendons under microCT. Ammonium molybdate shown to be effective at visualizing tendinous tissue under microCT. Mercury(II) chloride is good for visualizing individual muscle fascicles. However, it is highly toxic and generates hazardous waste. Mercury(II) chloride and ammonium orthomolybdate remained fixed in tissue over time.			
AgNO_4_ BaCl_2_, BaSO_4_ [31] Cs_2_CO_3_, CsCl, CsNO_3_ Cu(NO_3_)_2_, CuSO_4_ FeCl_3,_ FeSO_4_ KBr, KIO_3_, KMnO_4_ La(NO_3_)_3_ Na_2_MoO_4_ Pb(C_2_H_3_O_2_)_2_, C_6_H_8_O_7_Pb VOSO_4_ OsO_4_	Studied using a voxel size ranging from 23 µm to 40 µm except for barium sulfate, which was studied using a voxel size of 2.93 µm [31]. All these compounds were not shown to be effective for visualizing collagen microarchitecture with microCT. OsO_4_ is one of the first CESAs used for microCT. Binds well to adipose, but is highly toxic [12].			

## 3. Discussion

Although several options were identified in reviewing the relevant literature to find the optimal CESA for visualizing collagen microarchitecture using microCT, no one CESA is clearly optimal. Ultimately, CESA selection for the analysis of the microarchitecture of collagenous tissues depends on individual lab capabilities and imaging priorities.

Mono-WD POM provides the best contrast agent without tissue distortion, and more readily diffuses through tissues than other POMs; however, since it is not commercially available, it must be synthesized. This limitation may rule it out depending on lab capabilities and cost restrictions. PTA is backed by the most research, providing excellent visualization of collagen microarchitecture both using X-ray radiography and histologically; however, most studies report significant tissue distortion due its very low pH, as well as prolonged preparation time related to tissue preparation and staining prior to imaging. Less expensive options for CE-microCT of soft tissues include Lugol’s solution and sodium tungstate. Mercury(II) chloride is also relatively inexpensive; however, it generates hazardous compounds which can be difficult and expensive to dispose.

Although these CESAs are limited in their ability to provide sufficient contrast to see the orientation of collagen fibers, they diffuse through tissue relatively quickly (with the exception of mercury(II) chloride). Perhaps after some trial and error with different tissue staining procedures and scanning protocols, these contrast agents can provide sufficient image contrast to provide sufficient spatial resolution for the illustration of collagen fiber networks.

In addition to using CESAs, other methods in the reviewed literature were found to be useful for improving the signal-to-noise ratio using X-ray tube microCT scanners. Dudak et al. used a large photon-counting detector in their scans of various soft-tissue murine organs. They were able to achieve significant improvement in spatial and contrast resolution compared to standard charge-coupling detectors. They did so without the need for CESAs, using ethanol fixation alone [37]. This strategy significantly decreased the time needed for tissue preparation, and decreased the risk of tissue distortion by destructive CESAs. More research is needed to study the benefits of combining the use of a photon-counting detector with a CESA.

Two more ways of improving image contrast and spatial resolution with CT are the use of dual energy CT and phase contrast CT. Both methods work by adding a layer of data to be processed by the computer, creating more room for variability and thus more ways to show contrast. Dual energy X-ray (also called multispectral CT) scanning uses either two X-ray sources at different energy levels, or one X-ray source that can rapidly switch between energies, providing two distinct spectra of X-ray intensities to be picked up by the detector, thus allowing more distinction among tissue components both qualitatively and quantitatively [40]. This type of scanning is especially useful when using Lugol’s solution, as it helps to differentiate between different concentrations of the CESA within the specimen.

Phase contrast CT can use the intrinsic composition of the sample to present a new contrast mechanism in the image data. X-rays are photons and are subject to photonic interactions with matter, in this case with the electron cloud of elements which—like optical light—can refract photons and thus can refract X-rays [6,41]. In specific experimental conditions, this can be a contrast mechanism detected with a phase sensitive X-ray scanner. This requires the microCT scanner to be designed to detect the photon trajectory shift from the refraction. This is the simplest in synchrotron-generated monochromatic X-ray detectors, where the highly collimated X-ray beam is observed on off-axis detectors. As there is no compact (desktop) monochromatic X-ray source, other methods are used in micro CT systems. Benchtop microCTs have been developed to use X-ray phase-grating systems, edge-illuminated X-ray phase contrast, or diffraction-enhanced X-rays to produce and observe refracted X-rays [42,43,44,45]. These techniques use microCT poly-spectral X-ray sources, which are compact and isolate the phase-shifted X-rays for observation. Much of the output of the X-ray source is not refracted, and so is not observed for these phase-sensitive images. As such, scans with benchtop systems take more exposure time than attenuation (transmission) microCT images. In many cases, we are concerned about the specimen and its beam exposure transmission, and X-ray images in principle offer shorter specimen exposure times.

Another possible way to enhance contrast is with improvements in post-processing software capabilities. For example, ionic iodinated compounds and gadolinium-based compounds were found to stain non-collagen constituents of the soft tissues (GAGs and proteoglycans, respectively). Theoretically, the signals generated by these contrast agents could be isolated and used in post-processing to differentiate soft tissue constituents from collagen. Other image analysis and post-processing methods such as filtering, noise reduction, edge detection, and automatic tissue classification are being developed to provide higher quality images, especially with the use of additional data provided by dual energy CT [38]. Potentially, these and other post-processing software techniques could be used to digitally select and/or highlight the signal generated by stained collagen fibers to provide greater quantitative and qualitative detail.

A controlled comparison of the CESAs in Table 1 is the next step needed to find the optimal method for visualizing collagen microarchitecture using microCT. Objectives for this study could include a comparison of the contrast enhancement effects of different tissue preparation and staining methods, different scanning protocols and parameters, and different tissue or collagen types, similar to the CESA comparison performed by Pauwels et al. [12]. Once sufficient image contrast and spatial resolution are achieved, and standard methods for collagen fiber visualization using contrast-enhanced microCT are created, in vivo and ex vivo studies could be conducted to further understand the pathomechanics of collagen breakdown at a microscopic level. However, in vivo studies using microCT are much more limited due to microCTs increased sensitivity to motion artifacts and the need for relatively high concentrations of these toxic compounds.

With further microCT studies, the breakdown patterns of collagen under various types of stress can then be analyzed, as well as the pathophysiological reaction of fibroblasts to rebuild and strengthen the tissues against further degeneration or damage. MicroCT can be performed without dissecting or cutting the tissue, which has the advantage of providing an in situ illustration of the collagen construct free of potential tissue perturbation related to cutting or slicing the tissue. This additional knowledge could be used to improve our capabilities for characterizing tissue injury, for developing ways to prevent and treat soft-tissue injury, and for the fabrication of collagen-based tissue constructs that emulate or improve upon physiologic structures. Finally, while the CE agents discussed in this review offer benefits to visualizing soft tissue with microCT, there exists a need to discover new collagen-specific agents that are effective and gentle on tissues. Additional development of image processing software, and scanning contrast and spatial resolution improvements may lead to more frequent use of microCT by investigators for soft collagenous tissue analysis, for understanding normal tissue architecture, for understanding changes imparted by damage, and to compare engineered tissue or 3D-biofabricated tissues to their natural counterparts.

## 4. Materials and Methods

The literature search was performed using PubMed with the following search terms:

“X-ray microtomography” [MeSH Major Topic] AND (“Collagen” [MAJR] OR “collagen*” [All Fields] OR “soft tissue” [All Fields] OR “organ”.

At the time of this review, the search returned 270 results. Filtering the search by “Free Full Text” and “English” produced 149 results. From this pool, only articles with sufficiently detailed methods were included.

A total of 24 articles were selected for an in-depth comparison of the contrast agents used. From those 24 articles, a total of 48 contrast agents were identified for comparison. (See Table 1).

## Data Availability

Data sharing is not applicable.
